# An interfacial hydrogel compartment within a multicompartment tendon-to-bone scaffold influences cell behavior under cyclic tensile loading

**DOI:** 10.1093/rb/rbag042

**Published:** 2026-03-09

**Authors:** Kyle B Timmer, Megan L Killian, Brendan A C Harley

**Affiliations:** Department Chemical and Biomolecular Engineering, University of Illinois Urbana-Champaign, Urbana, IL 61801, USA; Department of Orthopaedic Surgery, University of Michigan Ann Arbor, Ann Arbor, MI 48109, USA; Department of Molecular and Integrative Physiology, University of Michigan Ann Arbor, Ann Arbor, MI 48109, USA; Department Chemical and Biomolecular Engineering, University of Illinois Urbana-Champaign, Urbana, IL 61801, USA; Cancer Center at Illinois, University of Illinois Urbana-Champaign, Urbana, IL 61801, USA; Carl R. Woese Institute for Genomic Biology, University of Illinois Urbana-Champaign, Urbana, IL 61801, USA

**Keywords:** rotator cuff enthesis, biomaterial, cyclic tensile loading, mesenchymal stem cells

## Abstract

Injuries to spatially graded insertional tissues such as the tendon-to-bone enthesis in the rotator cuff present unique challenges for regenerative engineering. To address these, tissue engineering approaches are increasingly considering the use of biomaterials that display spatially graded properties to mimic aspects of the tendon, bone and connecting fibrocartilage enthesis zones. Mechanical loading introduces an additional opportunity to locally deliver disparate mechanical signals to cells across a biomaterial that contains spatial changes in composition, structure or mechanical properties. Here, we demonstrate the significance of *in vitro* mechanical stimulation via a cyclic tensile strain bioreactor on progenitor cell activity in a collagen scaffold that contains tendon and bone-specific compartments linked by a continuous gelatin hydrogel interface. We demonstrate that inclusion of a hydrogel interfacial architecture modulates local patterns of strain across the biomaterial as well as differences in mesenchymal stem cell activity, secretion of pro-regenerative cytokines and expression of enthesis-associated genes. Further, we report region-specific shifts in gene expression in response to mechanical loading, the presence of a hydrogel interface and their cross-interaction. Broadly, these findings demonstrate the importance of considering mechanical stimulation when designing spatially graded biomaterials for the regeneration of interfacial tissue, such as the tendon-to-bone enthesis, providing insight into how environmental factors and material design can shape spatial and temporal trajectories of pro-regenerative activity.

## Graphical abstract

**Graphical Abstract:**
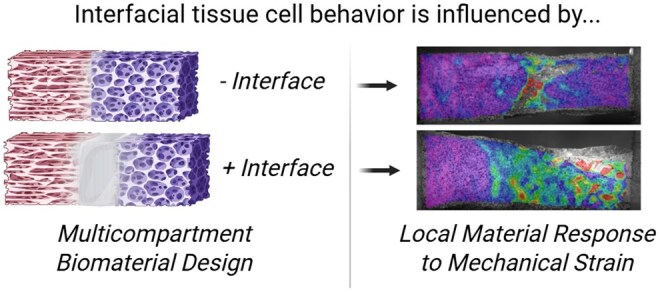

## Introduction

Biomaterials are a critical component of the tissue engineering triad. Their design often focuses on providing structural, compositional or mechanical cues to influence cell activity toward a specific tissue phenotype or behavior [1–4]. Interfacial tissue engineering requires considerations to replicate or promote regenerative healing of a transition that connects dissimilar tissues [5–8]. Two prominent considerations of interfacial tissue engineering are the replication of the native tissue’s anatomical properties as well as controlled cell behavior that mimics the interfacial tissue, such as gradients of cell type and secreted biomolecules [9, 10]. The fibrocartilaginous tendon-to-bone junction, also known as the enthesis, is among the most prominent. The supraspinatus enthesis of the rotator cuff is one such interface. Rotator cuff injuries represent one of the most common musculoskeletal disorders, affecting over a quarter of the population over the age of 60, with the prevalence of such injuries and subsequent surgical repair has been steadily growing [11–16]. Surgical reattachment can incur high rates of retear and failure, particularly with large tears (including other rotator cuff tendons) and in older patients [17–19]. These poor outcomes are often associated with a failure to regenerate the enthesis, which provides crucial mechanical support in transferring loads and reducing harmful strain concentrations between stiff, mineralized bone and soft, non-mineralized tendon [7, 20–22]. Load transfer occurs across a fibrocartilage zone that contains a gradation from unmineralized to mineralized fibrocartilage [23]. The enthesis harbors unique cell populations and matrix organization, such as high levels of collagen II, collagen X and aggrecan, and populations of fibrochondrocytes and hypertrophic chondrocytes [24]. This zonal organization of musculoskeletal interfaces suggests the need for biomaterials that mimic key transitions in not only extracellular matrix composition but also pro-regenerative or immunomodulatory cytokines, hypoxia and mechanical properties [25].

Mechanical loading is a critical factor responsible for maturation of the tendon-to-bone enthesis, affecting *in vivo* muscle volume, fibrocartilage development, collagen fiber alignment and mineralization [20, 26, 27]. On a cellular level, mechanical forces influence the expression and production of numerous cytokines, growth factors, transcription factors and extracellular matrix components [24, 28–31]. Loading also influences crucial healing outcomes following rotator cuff injury [32, 33]. From a regenerative engineering perspective, two critical aspects of mechanical loading must be considered. First, it is important to develop systems that improve our understanding of how bulk loading paradigms influence local cell activity across a spatially graded biomaterial, where applied stress or strain may not be monolithically distributed [34]. Second, biomaterials containing zonal transitions may be particularly affected by the emergence of local regions of strain or stress concentration, leading either to biomaterial failure or regions of excessive (or insufficient) levels of loading that negatively affect cell activity [35, 36]. Like the native enthesis, an engineered material must be able to functionally transfer loads across the tendon-to-bone transition, making possible the application of unique regions of mechanical stimulation while also avoiding the emergence of detrimental concentrations of local strain.

We have previously described methods to create porous collagen-glycosaminoglycan scaffolds via a lyophilization process [37, 38]. This work identified variants that can selectively promote human mesenchymal stem cell (hMSC) differentiation toward osteogenic (mineralized collagen scaffold) [39–41] or tenogenic (anisotropic non-mineralized collagen scaffold) [42] lineages without the need for exogenous factors. We also described a strategy to fabricate a biphasic scaffold with tendinous and osseous compartments that can promote hMSC differentiation toward tenogenic and osteogenic lineages in a spatially constrained manner [43, 44]. Yet while a cyclic tensile strain bioreactor can significantly improve tendinous activity, application of bulk levels of strain across the biphasic material induces significant strain concentrations at the interface between tendon and bone compartments [42, 45, 46]. To address this challenge, we recently described a triphasic multicompartment design, integrating the tendinous and osseous scaffold components via a polyethylene glycol (PEG) hydrogel interface that significantly reduced strain concentrations at the interface and increased construct toughness in response to tensile strain, both critical benchmarks for surgical practicality [47]. We more recently identified a thiolated gelatin (Gel-SH) hydrogel as a model enthesis material that supports hMSC viability and that presents a permissive environment for the emergence of fibrochondrogenic phenotypes in response to paracrine signals from hMSCs in tendon- and bone-specific scaffolds [48, 49].

Given the biological importance of mechanical cues in enthesis development and healing, as well as the mechanical benefits previously observed for tendon and bone-specific phenotypes in a model biphasic (tendon–bone) scaffold, here we evaluate hMSC activity across a triphasic (tendon-enthesis-bone) biomaterial containing a Gel-SH insertion in response to cyclic tensile strain. The objective of this effort is to describe the emergence of tendon-, bone- and enthesis-specific phenotypes for hMSCs seeded across the triphasic biomaterial, evaluating the effectiveness and impact of this mechanically motivated material design on the mechanoresponse of hMSCs. We hypothesized that the inclusion of a compliant hydrogel interface would reduce strain concentrations and aid in the promotion of a tendon-to-bone gradient of differentiation, given the difference in local strain magnitudes across the graded biomaterial. We report that the presence of a mechanically compliant hydrogel interface within a multicompartment tendon-to-bone scaffold alters metrics of hMSC metabolic activity and local shifts in transcriptomic activity in response to extended *in vitro* culture under cyclic tensile loading.

## Materials and methods

### Multicompartment scaffold formulation

#### Collagen-glycosaminoglycan precursor slurry preparation

Collagen-glycosaminoglycan suspensions were prepared by homogenizing purified fibrillar bovine collagen (Collagen Matrix, Inc #IPFC20N.01) and chondroitin sulfate sodium salt (Spectrum Chemical Mfg. Corp #C1610) within an acidic buffer solution using an ULTRA-TURRAX disperser fitted with an S25N-25G-ST Dispersing Tool (IKA Works) [39, 50–53]. Two variants of collagen-glycosaminoglycan precursor slurry were produced, as described in Table 1: a mineralized slurry homogenized in an acidic buffer (0.1456 M phosphoric acid, CAS: 7664-38-2, 7732-18-5, Fisher Scientific #A242; 0.037 M Calcium Hydroxide, CAS: 1305-62-0, Sigma-Aldrich), and a non-mineralized slurry homogenized in an acidic solution (0.05 M, CAS: 64-19-7, Fisher Scientific #A38-212). Homogenization was performed in a cooled, jacked vessel, and all slurries were stored at 4°C for at least 12 hours prior to use to maintain collagen integrity and allow for adequate hydration.

**Table 1:** Summary of precursor slurry variants.

	Collagen	Chondroitin sulfate	Additional salts
Mineralized	1.9w/v%	0.84w/v%	0.64w/v% Ca(NO_3_)_2_ · 4H_2_O 0.39w/v% Ca(OH)_2_
Non-mineralized	0.5w/v%	0.044w/v%	—

#### Gel-SH synthesis

Gelatin (type B, derived from bovine skin, CAS: 9000-70-8, Sigma-Aldrich #G9391) was thiolated as previously described by dissolving at 1w/v% in deionized water at 50°C and reacted with Traut’s reagent (2-iminothiolane-HCl, CAS: 4781-83-3, #PI26101 ThermoFisher Scientific Chemicals Inc.) at a 2:1 molar ratio of Traut’s to free amines on the gelatin backbone for 20 minutes followed by an additional 2 hours after reducing the solution pH to 5.0 [48, 49]. The product was purified via dialysis using 12–14 kD MWCO dialysis tubing (08-667E, Spectrum Spectra/Por 4 RC Dialysis Membrane Tubing, Fisher Scientific) against 5 mM HCl for 24 hours and 1 mM HCl for an additional 24 hours, after which the solution was frozen, lyophilized, and stored at −20°C.

#### Scaffold production

Multicompartment scaffolds were produced using custom molds made of polytetrafluoroethylene and copper [47, 49, 54]. Briefly, non-mineralized and mineralized slurries were pipetted into either end of the mold well using a 3D-printed divider, with the non-mineralized slurry against the well’s lone copper side. A 3.5 wt% Gel-SH hydrogel precursor solution 10 mM H_2_O_2_ (CAS 7722-84-1, H325-500, Fisher Scientific Inc.), 5 mM tyramine (T90344-5G, Aldrich Chemistry), 5 U/mL horseradish peroxidase (PI31490, Thermo Fisher Scientific Chemicals Inc.) in Dulbecco’s Phosphate Buffered saline (DPBS, 21-030-CV, Corning) was added between the two slurries [48]. The dividers were then removed for a period of 1 hour to allow the suspensions to mix at their interfaces. Molds were then transferred to a VirTis Genesis 25XL freeze-dryer and lyophilized through a previously established protocol: cooling to −10°C for 30 minutes, holding at −10°C for 2 hours, then sublimating the frozen constructs at 0°C and 0.2 Torr overnight or until satisfactory pressure differential was achieved. Scaffolds were trimmed post-lyophilization into desired dimensions (26 mm × 6.5 mm × 5 mm) using razor blades and cutting jigs.

#### Scaffold embedding, sterilization

Scaffolds for *in vitro* culture were embedded into custom 3D-printed polylactic acid (PLA) end blocks with a silicone compound (RTV615, Momentive Specialty Chemicals Inc.) and then cured at 60°C overnight using previously described methods [49]. Constructs were sterilized with ethylene oxide in a 12-hour cycle (AN74i Anprolene gas sterilizer, Andersen Sterilizers Inc.) [55–57].

#### Scaffold hydration, crosslinking

Embedded constructs were then hydrated and crosslinked under sterile conditions as previously described [46, 47, 54]. Briefly, materials were hydrated by soaking at room temperature in ethanol for 2 hours, followed by 1 hour in phosphate-buffered saline (PBS). Hydrated samples were then subjected to carbodiimide crosslinking for 90 minutes at 37°C via treatment with 1-ethyl-3-(3-dimethylaminopropyl) carbodiimide hydrochloride (EDAC, Sigma-Aldrich) and N-hydroxysulfosuccinimide (NHS, Sigma-Aldrich) at a 5:2:1 molar ratio of EDAC, NHS, and scaffold carboxyl groups, calculated as a function of pre-embedded scaffold mass [51]. Following crosslinking, scaffolds were washed in PBS and then left for 48 hours in MSC growth media consisting of 1% antibiotic-antimycotic solution (ThermoFisher Scientific) and 10% fetal bovine serum (Gemini Bio Products) in low glucose, glutamine-supplemented Dulbecco’s Modified Eagle Medium (DMEM, School of Chemical Sciences Cell Media Facility, University of Illinois Urbana-Champaign).

### Hydrated scaffold tensile testing

Scaffolds were mechanically tested under tensile load using a Psylotech Micro Test System and a 100 N load cell. Each sample was clamped in place and strained at a rate of 1 mm min^−1^ (approximately 4% strain min^−1^) until fracture with tensile elastic modulus (slope of the linear region), ultimate tensile stress, yield strain, and toughness calculated as previously described [47]. In addition, scaffolds were dusted with a layer of silicon carbide powder 240 (P280 grit, SIC-240-P1, Dace Technologies) and imaged during tensile testing using to visualize local strain across the biomaterial. Speckled images were analyzed using VIC 2D software (Correlated Solutions) to calculate local strain in the direction of displacement (ε_xx_) at each time point, with subset size and step size set at 39 and 6, respectively. Heat maps were generated to visualize local stress and strain across the specimens at each time point captured [47].

### Cyclic tensile strain bioreactor

**Figure 1:**
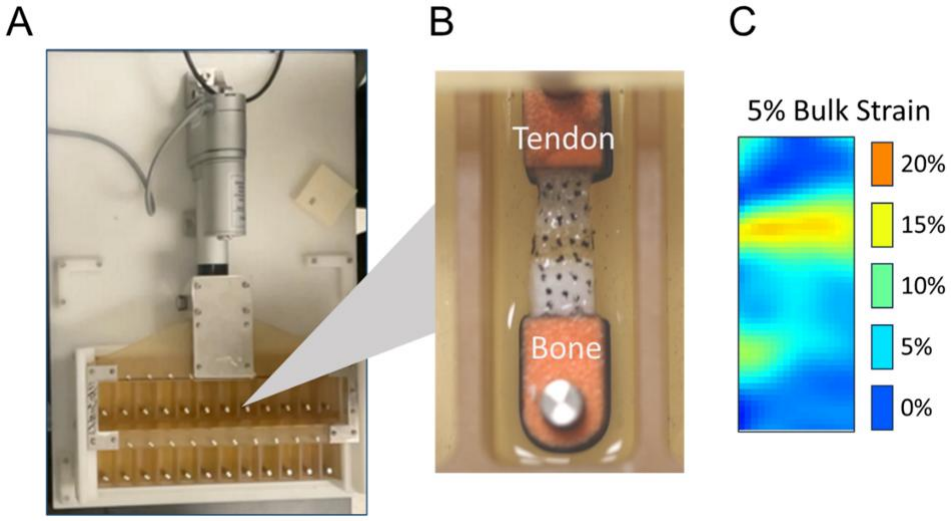
Cyclic tensile strain bioreactor setup and strain profile. (**A**) Bioreactor apparatus, (**B**) embedded scaffold with ink markers for digital image correlation and (**C**) MATLAB-calculated local strain profile at a 5% bulk strain, generated from images of the setup depicted in (**B**) within (**A**).

Scaffolds were assessed *in vitro* in a mechanically loaded environment using a custom bioreactor described in previous work [42, 46]. Embedded scaffolds were attached to loading posts fastened into the wells of the apparatus. Strain was applied by a sliding rake system, implemented by a linear motor actuator and controlled via an adapted C# program (Pololu Corp.). A static control apparatus with identical loading posts but no moving parts was used for non-loaded control scaffolds. The bioreactor used a previously validated loading profile: cyclic 5% bulk strain at 1 Hz for 10 minutes every 6 hours, designed to mimic physiological strain experienced by tendon/ligament and to maximize ERK1/2 activation [46, 58]. Frequency-based parameters were implemented based on conversion factors provided by the manufacturer, and displacement settings were confirmed via calipers, with settings correlated based on a standard curve (Supplementary Figure S1). The emergence of local strain profiles from bioreactor-induced tensile strain was confirmed via digital image correlation (DIC), obtaining images across the loading cycle using a Canon EOS 5DS R DSLR camera and analyzing resultant strain via an adapted MATLAB package, as previously described [49]. A depiction of this setup, the loaded material and the observed strain is provided in Figure 1.

### Cell culture and scaffold seeding

hMSCs (source: 20-year-old female donor, African American ethnicity, no known pre-existing conditions, Lot: 310263, RoosterBio, Inc.) on their fourth passage were thawed from liquid nitrogen storage and expanded in 2D culture under standard cell culture conditions (37°C, 5% CO_2_) using RoosterNourish^TM^-MSC (RoosterBio, Inc.). Following expansion, hMSCs were lifted and seeded onto scaffolds loaded into either the bioreactor or the static control apparatus using an established point seeding method [48]. Scaffolds were seeded with a total of 600 000 cells in 15 μL of media, delivered as 5-μL aliquots to each region (tendon, interface, bone), then incubated for 2 hours to allow infiltration at initial attachment. Following seeding, scaffolds were submerged in mesenchymal stem cell growth media which was changed every 3 days.

### Cellular metabolic activity analysis

Metabolic activity (*n* = 3–6) of hMSCs in each scaffold zone was measured using an alamarBlue^TM^ activity assay (Thermo Fisher). Scaffolds were removed from the media, and a cutting guide was used to cut the graded construct into tendon, interfacial, and bone regions. Each region was incubated individually for 90 minutes on a shaker while suspended in a 9:1 volume ratio of mesenchymal stem cell growth media and alamarBlue^TM^ (ThermoFisher Scientific). The media solution was removed and measured in triplicate on a Tecan F200 spectrophotometer at 540/580 nm fluorescence. A standard curve using known cell numbers at experimental day 0 was generated to correlate intensity readings with cell number.

### RNA isolation and evaluation

As outlined in previous work, RNA was isolated from cells either by RNAqueous-Micro Total Isolation Kit (cell solutions, ThermoFisher Scientific) as per manufacturer instructions or by Trizol-chloroform extraction followed by DNase 1 treatment and purification (cells in scaffolds, RNA Clean & Concentrator Kit, Zymo Research) [54]. RNA was quantified by measuring UV-Vis absorbance via a NanoDrop One^C^ spectrophotometer (ThermoFisher Scientific) and subsequently stored at −80°C until further analysis. Gene expression analysis was conducted via reverse transcription–quantitative polymerase chain reaction (RT-qPCR) (*n* = 4–6). Isolated RNA was first converted to cDNA (QuantiTect Reverse Transcription Kit, Qiagen; S100 Bio-Rad Thermal Cycler). PCR reactions were next run with 45 ng cDNA per well and manufacturer-recommended concentrations of TaqMan Fast Advanced Master Mix and the following TaqMan primers (Table 2).

**Table 2:** TaqMan probes used for gene expression assays.

Gene name	Taqman assay ID
*RPL13a*	Hs04194366_g1
*COL1A1*	Hs00164004_m1
*COMP*	Hs00164359_m1
*IL6*	Hs00923996_m1
*MMP3*	Hs00968305_m1
*SCX*	Hs03054634_g1
*SOX9*	Hs00165814_m1
*SPP1*	Hs00959010_m1
*RUNX2*	*Hs01047973_m1*

All reactions were run in duplicate using a QuantStudio^TM^ 7 Real-Time PCR System and converted into fold change data using the ΔΔCT method, normalizing each sample to RNA isolated from unseeded hMSCs at experimental day 0 as well as to expression of *RPL13a*, a housekeeping gene demonstrated as reliable and stable for MSC culture toward osteogenesis, adipogenesis, chondrogenesis and tenogenesis [59–62].

### Secreted protein quantification

Cell culture media introduced on experimental day 4 and removed on experimental day 7 were pooled from available samples (*n* = 3–5) for each of the four experimental conditions (triphasic versus biphasic; loaded versus static) and assessed via semi-quantitative membrane-based cytokine array. Secreted protein release was measured via a Proteome Profiler Human XL Cytokine Array Kit (#ARY022B, R&D Systems), run according to manufacturer recommendation using previously described methods [54]. Differences in culture media introduced on experimental day 4 and removed on experimental day 7 were compared between the four groups. Dot intensity was quantified using ImageJ by measuring pixel intensity over a defined area, and raw intensities for each sample were normalized against the average intensity of positive controls. Each membrane contained two blots per cytokine, which were both individually analyzed and plotted.

### Statistical analysis and data visualization

All statistical analysis was conducted using R (version 4.3.3) through the program RStudio (version 2024.12.1 + 563). Outliers were defined as data points falling outside the dataset’s 1.5 interquartile range (IQR), and all outliers were removed prior to statistical analysis (though they are still included in data visualization). Following outlier screening, data were assessed for normality (Shapiro–Wilk test) and homogeneity of variance (Levene test), with the threshold for significance set at 0.05. Following characterization of each dataset, significance testing was conducted based on these results, with data following a normal distribution and equal variance tested for significance via analysis of variance (ANOVA) and, if applicable, a Tukey’s post hoc test. Data with neither of these properties were tested for significance via Welch’s Heteroscedastic F-test (trimmed means and winsorized variances) and a Games–Howell post hoc. Separate tests were also used for normally distributed data without equal variance (Welch’s ANOVA, Games–Howell) and for data with equal variance but without a normal distribution [Kruskal–Wallis, Dunn (Benjamini–Hochberg)].

Figures were created using the program OriginPro 2024 edition. Box-and-whisker plots generated indicate the sample median via a line, the 0.25–0.75 percentile range via the box, and the ±1.5 IQR via the whiskers. Points on the graph depict data outside of the IQR range, considered outliers. An asterisk, “*”, indicates statistical significance of a particular group. If alone, it indicates significance from all other groups of its subgroup. If included with branches, it indicates significance specifically between two members of a subgroup. For data sets with many subgroups, letters are instead used to depict significance. Here, matching assigned letters indicates groups that are not statistically significant from each other. Groups without a matching letter are significantly different from one another.

## Results

### A compliant interfacial zone alters scaffold resilience to tensile loading

We first evaluated the mechanical properties of hydrated multicompartment (tendon-to-bone) scaffolds with or without an integrated Gel-SH hydrogel interface in response to uniaxial tensile loading. Scaffolds containing an interfacial Gel-SH hydrogel demonstrated significantly (*P* < 0.05) reduced elastic modulus and toughness, though the inclusion of the hydrogel interface also resulted in significantly greater capacity for strain before fracture (Figure 1A). DIC also confirmed that the inclusion of a hydrogel enthesis zone resulted in significantly reduced high-intensity strain concentrations, particularly at the tendon–bone interface, with strain instead distributed more uniformly across the tendinous compartment. (Figure 1B).

### A compliant interfacial zone alters cell stress behavior in response to mechanical cues

**Figure 2:**
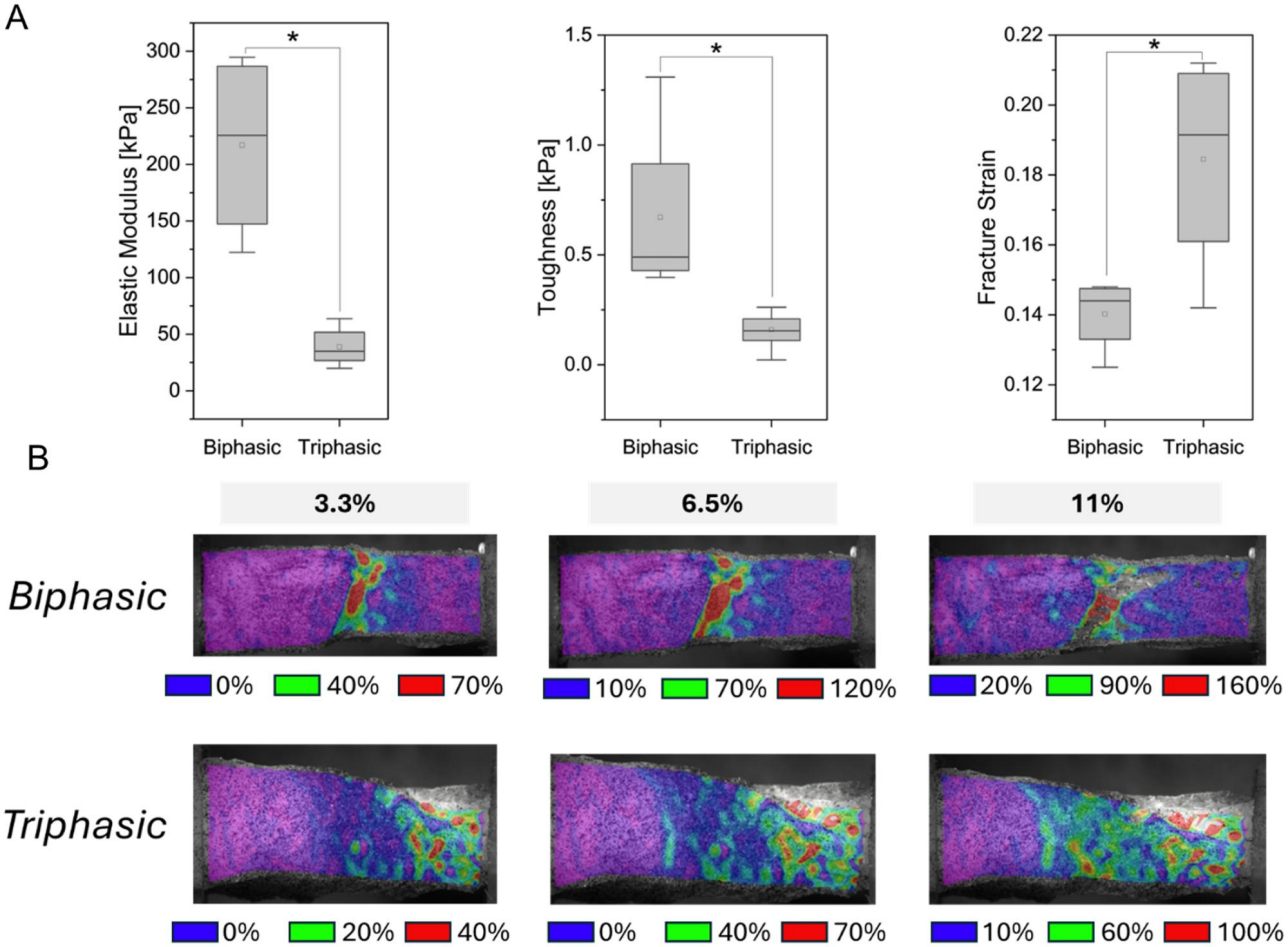
Bulk and local mechanical properties of multicompartment scaffolds. Scaffold samples (*n* = 4–12) were subjected to unidirectional tensile testing to generate stress-strain curves and local strain heat maps via digital image correlation. (**A**) Bulk material properties of elastic modulus, toughness, and fracture strain were calculated from stress-strain information. *: p < 0.05. (**B**) Digital image correlation heat maps of multicompartment scaffolds depicting local strain concentrations at three different bulk strain values. Approximate magnitude of low, medium, and high local strain are provided as a key for each condition.

hMSC activity across the multicompartment scaffold was evaluated after 7 days with or without exposure to cyclic tensile loading (Figure 2). Broadly, while no significant cross-interaction between scaffold region and loading condition was observed with triphasic scaffolds, a very significant (*P* < 0.01) cross-interaction was observed for these variables in biphasic scaffolds. For the biphasic group, loading resulted in a significant increase in metabolic activity within the tendon region and a strong trend (*P* < 0.10) of reduced metabolic activity within the interfacial region.

**Figure 3:**
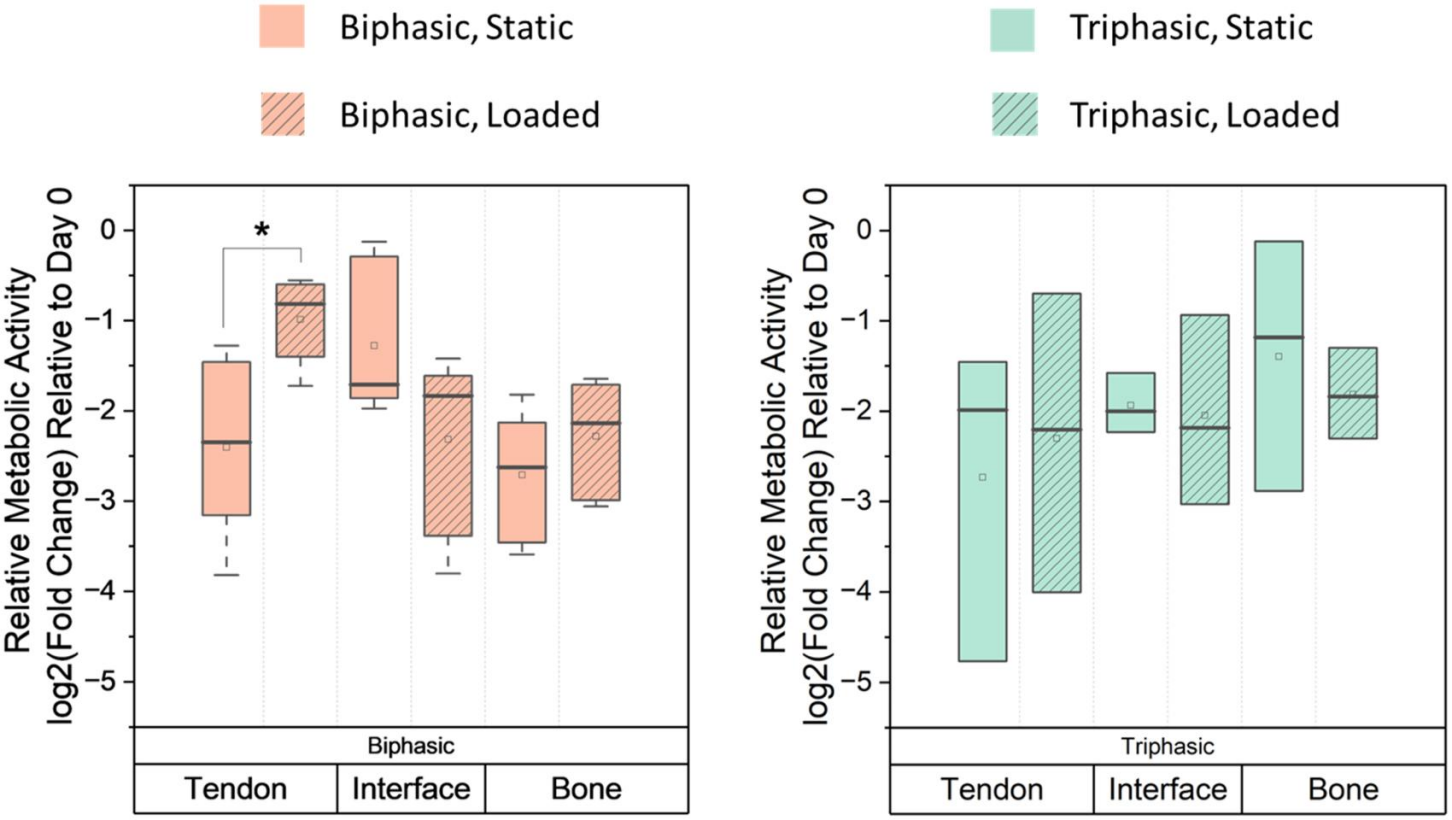
Metabolic activity of hMSCs in response to a compliant hydrogel interface and/or a mechanically loaded environment. Metabolic activity of cell-seeded, isolated multicompartment sections (*n* = 3–5) assessed after 7 days of culture. *: p < 0.05.

Additionally, we investigated the secretion of hMSC cytokines within these four groups during the final 72 hours of culture via a cytokine array of human cytokines and chemokines associated with inflammation, angiogenesis, and musculoskeletal development. Notably, mechanical loading appeared to reduce expression of secreted factors Dkk-1, IGFBP-2, PTX3, and VEGF while increasing secretion of IL-8 (Figure 3). Further, while not affected by mechanical loading, osteoclastogenesis inhibitor CHI3L1 was expressed at higher levels by cells in triphasic scaffolds relative to biphasic.

### A compliant interfacial zone alters cellular tendon-to-bone behavior in a mechanically loaded environment

**Figure 4:**
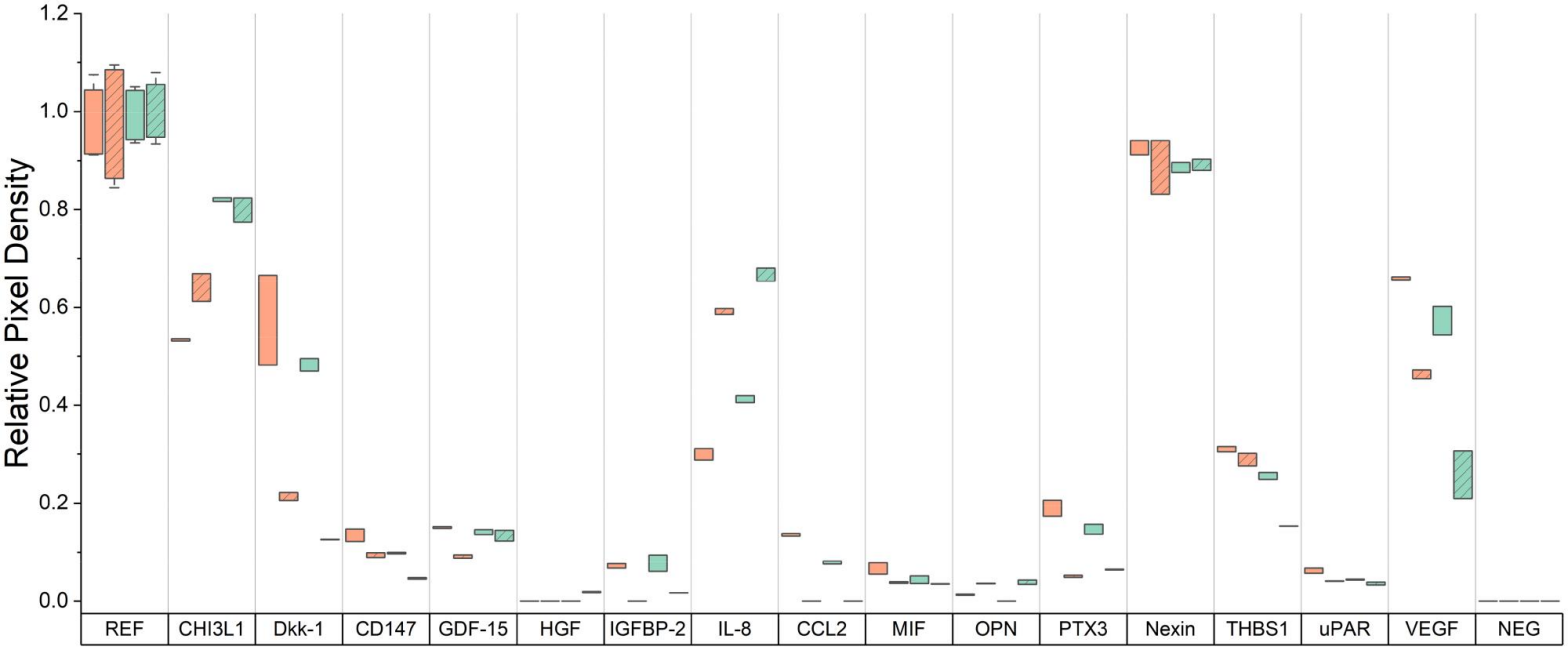
hMSC secretion profiles in response to scaffold type and cyclic tensile loading. Cytokine presence within pooled media samples (*n = *2 replicates per cytokine; *n = *1 membrane per experimental group) taken in the final 72 hours of cell culture (days 4–7). Bars depict the range of values generated from dot blot analysis (cytokine: *n* = 2; reference: *n* = 6).

**Figure 5:**
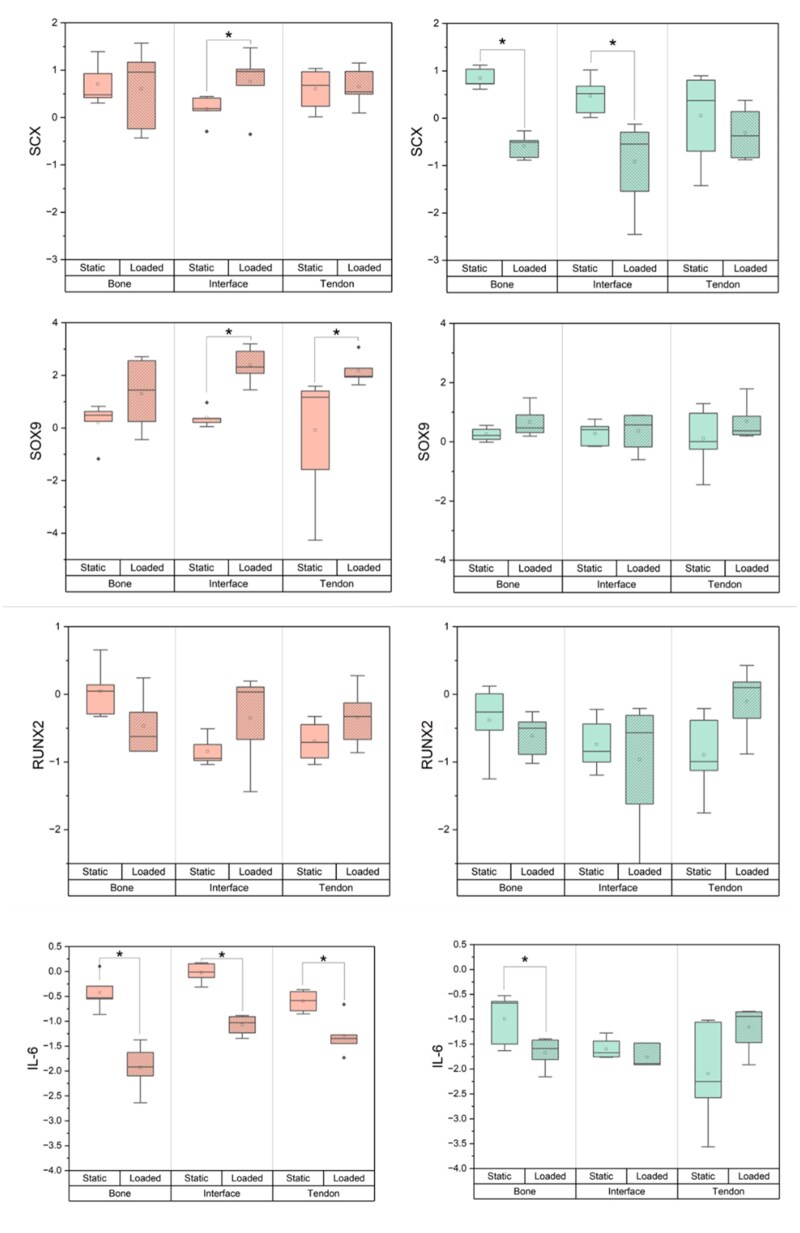
Region-specific gene expression profiles of hMSCs within multicompartment scaffolds in response to scaffold type and cyclic tensile loading. Samples (*n* = 3–5) isolated from hMSCs within 2mm sections of each multicompartment region after 7 days for a variety of enthesis-associated genes. Scaffold types (biphasic, triphasic) and environments (loaded, static) were compared. Graphs depict log2(Fold Change) from a Day 0 control. *: p < 0.05.

**Figure 6:**
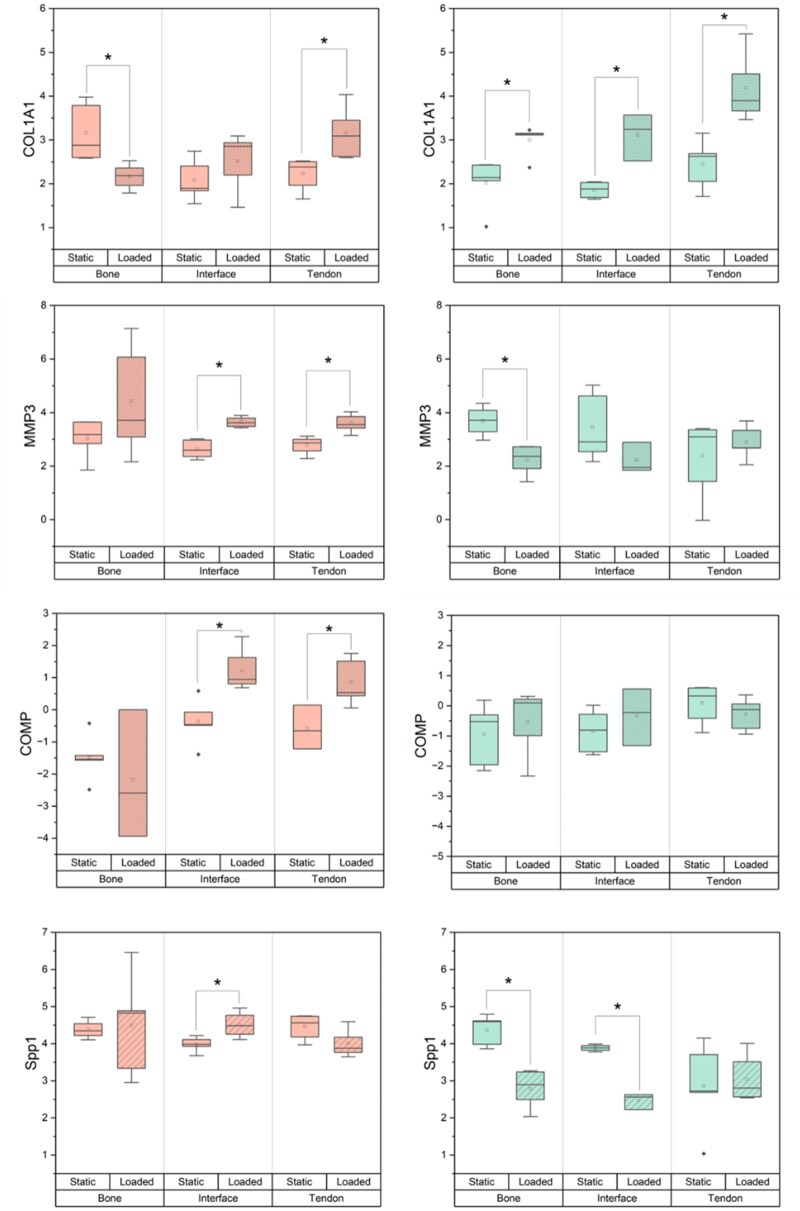
Additional region-specific gene expression profiles of hMSCs within multicompartment scaffolds in response to scaffold type and cyclic tensile loading. Samples (*n* = 3–5) isolated from hMSCs within 2mm sections of each multicompartment region after 7 days for a variety of enthesis-associated genes. Scaffold types (biphasic, triphasic) and environments (loaded, static) were compared. Graphs depict log2(Fold Change) from a Day 0 control. *: p < 0.05.

Finally, we assessed cellular gene expression over the 7 days of culture to identify differences associated with osteotendinous development. We first identified differences between biphasic and triphasic scaffolds in static culture (Figures 4, 5, and 6). The inclusion of an interfacial hydrogel in static conditions reduced the expression of osteogenic marker *SPP1* in the tendon region as well as *COL1A1* expression in the bone. Further, expression of the inflammatory cytokine gene *IL6* was significantly reduced across all regions of the static triphasic group relative to the static biphasic. hMSCs broadly expressed regional differences in *RUNX2, COL1A1* and *IL6* under static conditions.

Interestingly, the application of cyclic tensile strain significantly altered gene expression patterns across both scaffolds. In triphasic scaffolds subjected to loading, hMSCs significantly downregulated *SCX* in all regions, significantly upregulated *COL1A1* in all regions, and significantly downregulated *SPP1* in bone and interfacial regions. Trends of expression change were additionally observed in *MMP3, IL6* and *RUNX2*. These results were notably distinct from the biphasic response. While similar trends were observed for some genes, such as the expression of *RUNX2*, hMSCs in the loaded biphasic significantly upregulated *SOX9* and *MMP3* across all regions, as well as *SCX* and *COMP* within the tendon and interface. Further, *COL1A1* in the bone region was downregulated in response to loading within the biphasic scaffold. Broadly, scaffolds subjected to cyclic tensile strain displayed region-specific differential expressions of *COL1, COMP* and *IL6*.

## Discussion

Poor healing outcomes in rotator cuff repair, often stemming from an inability to regenerate the native tendon-to-bone enthesis, highlight the need for advances in interfacial tissue engineering. Here, this work considered the modification of a spatially graded biomaterial with discrete tendon and bone-specific compartments to now contain a compliant hydrogel zone connecting these two scaffold compartments. And further, what the role of cyclic tensile strain is on the emergence of pro-enthesis phenotypes in an *in vitro* bioreactor study. The application of cyclic tensile loading has previously been shown to alter cell behavior within a wide range of engineered 3D constructs (collagen, fibrin, PEG, gelatin) for improvement of tenogenic, chondrogenic, fibrochondrogenic, and enthesis-associated properties [63–68]. Additionally, while mechanical loading is highly relevant in the broad context of musculoskeletal tissues, the use of a spatially graded biomaterial offers the potential to locally tune the degree of mechanoactivation at discrete points across a multicompartment material. Depending on the structure, relative size and other physical properties of the engineered hard–soft interface, a material will present significant differences not only in bulk mechanical properties (elastic modulus, toughness, etc.) but also in profiles of local strain concentration, occurring at much smaller scales [69]. Cells are extremely sensitive to even minute mechanical forces and strains occurring due to matrix deformation or fluid flow, and such signals can significantly influence stem cell behavior and differentiation [70–72].

We first demonstrated fundamental differences in material properties associated with the inclusion of a Gel-SH hydrogel interface. While we have previously described many variants of this multicompartment scaffold design and the individual scaffold and hydrogel components [44, 46–49], including visualization of macro/microstructure and compressive/tensile moduli, this work is our first to directly compare our biphasic scaffold with our most recent, gelatin-based triphasic scaffold. The inclusion of a transitional gelatin hydrogel region significantly bolstered the amount of strain a multicompartment scaffold could withstand before fracture; however, the tradeoff of this benefit was a reduction in bulk elastic modulus and toughness of the overall construct. However, the ability to withstand significant levels of strain before fracture is a critical element of the native enthesis [73, 74]. DIC analysis of biphasic (tendon–bone) or triphasic (containing a hydrogel interface between tendon and bone compartment) highlighted further differences. While biphasic scaffolds experience significantly increased strain concentrations at the interface between tendon and bone, also a characteristic of improperly healed rotator cuffs subject to re-failure [75], this strain concentration was not observed in triphasic biomaterials containing the hydrogel enthesis zone. Together these results provide strong evidence that the inclusion of a Gel-SH hydrogel region not only results in better mimicry of the native enthesis function but also could significantly alter stem cell mechanotransduction at a local or region-specific level.

We subsequently examined the influence of local scaffold properties and the application of cyclic tensile strain on local and global changes in hMSC activity. We assessed metabolic activity and gene expression from discrete zones across the construct (tendon, enthesis, bone). In a biphasic scaffold, metabolic activity was significantly affected by cyclic loading, with increased activity with loading in the tendon region and a notable reduction (*P* < 0.10) of metabolic activity in the interfacial zone, which also displayed significantly increased levels of strain. In contrast, in triphasic scaffolds, we did not observe significant differences in metabolic activity between static and loaded groups, likely resulting from the combination of higher fracture strains (less material damage under 5% strain) and reduced local strain concentrations observed in the triphasic scaffold. However, cell secretion behavior was noticeably affected by loading in both scaffold groups, indicating a cellular response to the changed environment. In both groups, cells responded to cyclic tensile loading through reduced secretion of Wnt signaling pathway agonist DKK1 [76, 77], reduced secretion of IGF system regulator IGFBP2 [78–80], and altered secretion of pro-inflammation-associated cytokines (upregulation of IL8 [81], downregulation of PTX3 [82–84]). Further, the heightened secretion of CHI3L1, an osteoclastogenesis inhibitor, in the triphasic scaffold specifically could indicate a stronger pro-osteogenic environment, particularly when paired with the reduced PTX3, also an osteoclastogenesis promoter, observed with loading [83, 85]. Altogether, secretome analysis indicates a significant response in cell behavior to a cyclic loading regime, providing potential benefits toward pro-osteogenic and inflammatory responses.

Next, we considered the impact of scaffold mechanical loading on the emergence of a tendon-to-bone phenotype. Considering gene expression analysis, the behavior of *COL1A1* showcases the complexity of multicompartment biomaterials. *COL1A1* expression was significantly influenced by environment (static versus loaded), by scaffold type (biphasic versus triphasic), and by scaffold region (tendon, interface or bone), with extremely significant (*P* < 0.001) cross-interactions between loading environment and scaffold type as well as between loading type and scaffold region. These results coincide with the consensus that mechanical loading can increase the production of collagen and highlight not only the importance of each of these considerations but also the degree to which they can influence one another [29]. Other genes, including *SOX9, SCX, IL6* and *SPP1*, were also significantly affected by both environment and scaffold type. In a biphasic scaffold, the increase of *SOX9* and decrease of *IL6* expressions demonstrate significant impacts for mechanical loading on both chondrogenic and inflammatory behavior of hMSCs. Further, despite general trends of upregulation for *SPP1* and *SCX* in biphasic scaffolds as a response to mechanical loading, in a triphasic scaffold, these genes were significantly downregulated in response. These differences, as well as a significant reduction of *MMP3* in loaded triphasic scaffolds relative to loaded biphasic scaffolds, demonstrate that the inclusion of an interfacial hydrogel does not merely enhance or reduce previously seen effects but instead can completely reshape cell response to mechanical stimuli. As we have previously hypothesized, these results broadly demonstrate the importance of interaction between multicompartment material regions: changing an interface fundamentally alters the material not just mechanically but biologically. Future efforts will focus on exploring new loading regimes and more precise spatial investigation to better understand the mechanisms behind these interactions and the functional changes they can promote. While the current profile was selected based on previously established work for biomaterial *in vitro* culture and tendon-associated behavior [42, 86], the rotator cuff and broadly the musculoskeletal system are subjected to a vast number of loading systems, each with its own intensities, frequencies and patterns. Through this variety, we have a vast space to explore in further elucidating the relationships between cell behavior, material response and loading regime.

Finally, the significant differences in *IL6* expression between the triphasic and biphasic scaffolds motivate greater investigation into cellular inflammation and stress responses, particularly in response to mechanical loading. Ongoing work is focused on assessing functional secretion of human cytokines and chemokines, particularly those that could be associated with inflammation and cell stress, through quantitative ELISA analysis informed by the behavior observed via cytokine array here. This may reveal greater differences between the triphasic and biphasic scaffold environments, particularly in response to potentially harmful strain concentrations. Future work building on this study will consider the effects of longer-term tensile strain on pro-regenerative cell activities, providing the long-term impact of multi-phase collagen scaffolds on hMSC viability, differentiation, and overall regenerative efficacy for tendon-to-bone healing. Further, bioreactor–biomaterial systems such as this may offer advantages for future mechanistic investigation of processes underlying entheseal development, injury and regeneration. Perspectives on the mechanobiology of entheseal development [87] as well as past investigation using tendon-specific biomaterials in this cyclic tensile bioreactor [45, 46] suggest the need to explore a range of mechanosensitive pathways (e.g. YAP/TAZ, integrin signaling). Such transitional tissue engineering platforms may also offer an advantage over transcriptional or epigenetic studies using traditional *in vivo* models due to the often very limited number of cells that reside within a matrix-dense tissue *in vivo*. Broadly, the convergence of spatially graded biomaterials with cyclic strain bioreactors offers the opportunity for significant future growth. Through this and future analyses, we aim to build a broader understanding of cellular response to region-specific loading regimes, informing material design for future interfacial tissue engineering efforts.

## Conclusions

Here, we report the influence of including a compliant hydrogel interface into a spatially graded biomaterial for tendon-to-bone tissue engineering applications. The inclusion of a Gel-SH region to form a triphasic scaffold significantly increased the mechanical properties of the bulk scaffold, specifically leading to increased strain at failure and reduced strain concentrations during cyclic tensile loading relative to a biphasic biomaterial that did not contain the protective interface. This modification significantly altered MSC responses across the multicompartment material in response to *in vitro* cyclic tensile loading. Gene expression analysis further demonstrated these differences, highlighting significant changes in cell behavior depending on loading and scaffold type (*COL1A1, SOX9, SCX, IL6, SPP1*) as well as by scaffold region (*COMP*), demonstrating significant cross-interaction between these variables. Together, these results highlight the necessity of considering incorporating mechanical loading and cellular mechanotransduction when developing interfacial tissue-engineered materials.

## Supplementary Material

rbag042_Supplementary_Data

## Data Availability

Raw data and processed data will be made available upon request.
