# Supplementary material for: An interfacial hydrogel compartment within a multicompartment tendon-to-bone scaffold influences cell behavior under cyclic tensile loading
Source: Regen Biomater. 2026 Mar 9;13:rbag042. doi: 10.1093/rb/rbag042 (PMC13091616; doi:10.1093/rb/rbag042)
Supplement: rbag042_Supplementary_Data [file rbag042_supplementary_data.docx]

# Supplementary Materials for

# **An interfacial hydrogel compartment within a multicompartment tendon-to-bone scaffold influences cell behavior under cyclic tensile loading**

**Kyle B. Timmer^1^, Megan L. Killian^4,5^, Brendan A.C. Harley^1,2,3^**

^1^ Dept. Chemical and Biomolecular Engineering

^2^ Cancer Center at Illinois

^3^ Carl R. Woese Institute for Genomic Biology

University of Illinois Urbana-Champaign

Urbana, IL 61801

^4^ Department of Orthopaedic Surgery

^5^ Department of Molecular and Integrative Physiology

University of Michigan Ann Arbor

Ann Arbor, Michigan 48109

**Corresponding Author:**

B.A.C. Harley

Dept. of Chemical and Biomolecular Engineering

Cancer Center at Illinois

Carl R. Woese Institute for Genomic Biology

University of Illinois at Urbana-Champaign

110 Roger Adams Laboratory

600 S. Mathews Ave.

Urbana, IL 61801

Phone: (217) 244-7112

Fax: (217) 333-5052

e-mail: [bharley@illinois.edu](mailto:bharley@illinois.edu)


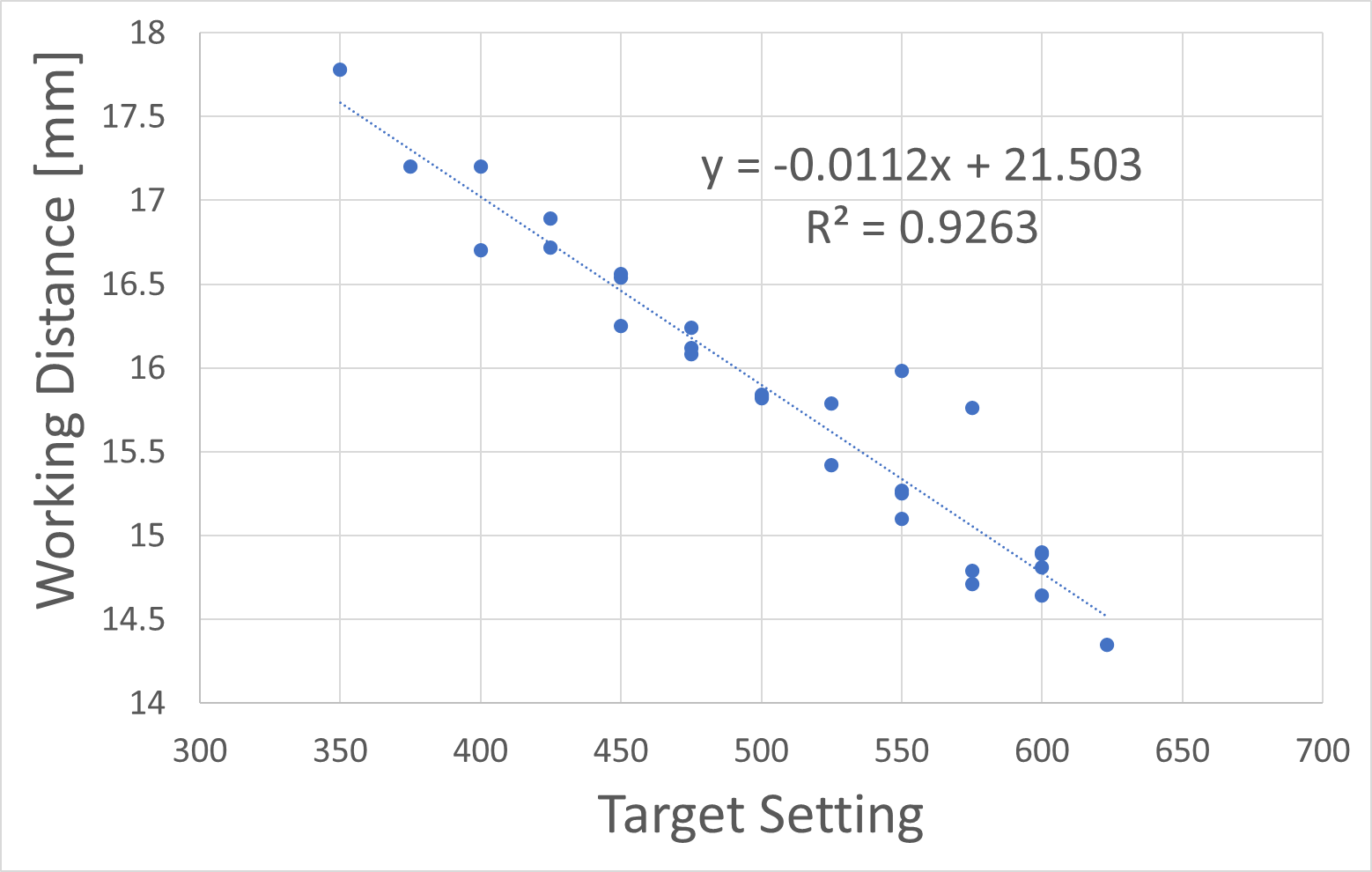


**Figure S1. Calibration curve for bioreactor displacement.** Distances measured via calipers and adjusted to represent working distance, defined as the distance between endblocks. Actuator was reset to 0 between each test to eliminate potential interference or bias.
